# Breastfeeding in the Context of Trauma and Previous Psychological Experiences: A Narrative Review

**DOI:** 10.3390/nu18030455

**Published:** 2026-01-30

**Authors:** Aleksandra Purkiewicz, Kamila J. Regin, Renata Pietrzak-Fiećko

**Affiliations:** 1 Department of Commodity Science and Food Analysis, Faculty of Food Science, University of Warmia and Mazury in Olsztyn, Plac Cieszyński 1, 10-720 Olsztyn, Poland; renap@uwm.edu.pl; 2 Department of Rehabilitation and Orthopedics, School of Medicine, Collegium Medicum, University of Warmia and Mazury in Olsztyn, 10-726 Olsztyn, Poland; kamila.regin@uwm.edu.pl

**Keywords:** lactation, trauma, psychology of breastfeeding, mother-child bond

## Abstract

Breastfeeding is a complex biopsychosocial process influenced not only by biological mechanisms but also by a woman’s previous psychological experiences and past traumas. The aim of this review was to analyze current research on the impact of early traumatic experiences, perinatal trauma, psychological difficulties, and previous interpersonal stressors on the initiation, continuation, and emotional course of breastfeeding. Women with a history of trauma are more likely to struggle with emotional regulation difficulties, increased stress, depressed mood, and problems bonding with their child. These factors translate into an increased risk of discontinuing lactation, discomfort during feeding, and reduced self-esteem regarding maternal competence. The literature also emphasizes the role of psychological and social support, which can help mothers cope with emotional tension and promote a positive breastfeeding experience. Consideration of the mother’s previous psychological and traumatic experiences is crucial for a more complete understanding of lactation difficulties and the development of effective forms of support for women in the perinatal period.

## 1. Introduction

Breastfeeding is the most recommended method of meeting a child’s nutritional needs, with beneficial effects for both mother and infant [1]. It is reported that breastfeeding promotes cognitive development in children, protects against infections, and reduces the risk of obesity in the first years of life [2,3,4]. Breastfeeding mothers are less likely to develop chronic diseases such as type 2 diabetes, ovarian cancer, breast cancer, and cardiovascular disease [5]. In addition, breastfeeding plays a key role in the proper mental development of the mother and child, promoting the formation of a unique emotional bond between them [6].

The World Health Organization (WHO) and the United Nations Children’s Emergency Fund (UNICEF) recommend starting breastfeeding immediately after birth and feeding exclusively for at least 6 months, continuing breastfeeding until at least 2 years of age with appropriate complementary foods [7]. Despite the numerous proven benefits of breastfeeding, according to WHO data from 2023, only 44% of infants aged 0–6 months worldwide are exclusively breastfed [8].

Breast milk provides all the necessary nutrients in proportions adapted to the needs of a developing organism, including high-quality complete protein, long-chain polyunsaturated fatty acids (LCPUFAs), lactose, vitamins, minerals, and unique bioactive compounds, including immunoglobulins, lactoferrin, lysozyme, and human milk oligosaccharides (HMOs), as well as numerous immune cells that support the development of the infant’s immune system [6,9]. It has been shown that breastfed children have a lower incidence of infections, upper and lower respiratory tract infections, otitis media, gastrointestinal problems, and urinary tract infections [10]. In addition, breastfeeding provides many long-term benefits for the child, including protection against obesity, diabetes, allergies, and chronic diseases in adulthood [1]. Breastfeeding plays an important role not only in terms of nutrition but also psychologically. This process promotes the formation of a strong emotional bond between mother and child, based on physical closeness, touch, and mutual eye contact [11]. Building this relationship is crucial for the child’s proper emotional development, sense of security, and stability in the relationship with the caregiver [6]. For the mother, breastfeeding is an important part of adapting to her new role, strengthening her sense of self-efficacy and parental competence [12]. Numerous studies indicate that breastfeeding women have lower levels of postpartum stress and a lower risk of postpartum depression, which is associated, among other things, with the action of oxytocin—a hormone secreted during breastfeeding, responsible for feelings of calm, relaxation, and attachment [13,14]. Other important benefits of breastfeeding for mothers include protection against the development of chronic diseases and a lower risk of infection. It has been shown that longer breastfeeding duration is associated with a reduced risk of breast and ovarian cancer, type 2 diabetes, and cardiovascular disease [15]. In addition, breastfeeding promotes proper uterine involution after childbirth, which reduces the risk of postpartum bleeding and infections [16]. The lactation process also increases the body’s energy expenditure, which may contribute to a faster return to pre-pregnancy weight [17].

Economic and practical benefits are also important. Breastfeeding is a cheaper solution compared to feeding infants with infant formulas—it does not require the purchase of preparations and feeding accessories [18]. Breast milk is always available, at the right temperature, and can be used without the need for drinking water, which is particularly important in conditions of limited access to sanitary facilities [19,20]. As a result, breastfeeding can be considered a process of multidimensional significance, combining biological and psychological benefits, for both mother and child [21]. Breastfeeding is an optimal, safe, and economical way to feed infants, providing measurable health benefits for both mother and child [22].

There is comparatively little attention paid to the difficulties faced by breastfeeding mothers and the psychological problems that may accompany this process [23]. These challenges include not only physical pain and discomfort, social pressure related to expectations about breastfeeding, or feelings of guilt in case of failure but also deeper psychological factors [23,24]. In some cases, breastfeeding may be associated with reliving past traumas, experiences of abuse, or difficult emotions that affect the ability to bond with the baby and the perception of the breastfeeding process itself. In addition, previous psychological experiences, such as anxiety disorders, depression, or body image issues, can significantly affect the course and emotional dimension of lactation [25]. Therefore, in addition to promoting the benefits of breastfeeding, it is important to consider the psychological aspects of this process and to provide support to women who experience emotional or psychological difficulties. A woman’s decision to stop breastfeeding can be determined by numerous factors. It is crucial to understand women’s experiences of breastfeeding. In the context of breastfeeding, difficulties are considered in the context of biological, psychological, and social factors [23]. Difficulties in the feeding process can directly influence attitudes and practices related to breastfeeding and determine mothers’ decisions to stop breastfeeding [26].

Although the relationship between breastfeeding and maternal mental health has been the subject of numerous studies [11,26,27,28], a life-course perspective that considers traumatic experiences remains relatively underrepresented in the literature. This review expands on existing approaches by incorporating traumatic experiences from different stages of life in the analysis of the relationship between mothers’ mental health and breastfeeding. The analysis of the relationship between mothers’ mental health and breastfeeding a trauma-informed perspective, which has been addressed only to a limited extent in reviews of this area to date. This literature review presents the current state of knowledge on the impact of traumatic experiences and previous psychological difficulties on the breastfeeding process. The aim of the study is to show how psychological factors such as trauma, postpartum depression, anxiety, or negative past experiences can shape attitudes toward breastfeeding, influence its initiation and maintenance, and affect the mother’s subjective experience. The review also aims to highlight the importance of adequate psychological and systemic support for breastfeeding women, especially those who have experienced trauma or other emotional burdens in the past. The review is based on a conceptual framework that includes life-course perspective and traumatic experiences, which facilitates a coherent analysis of the relationship between mothers’ mental health and breastfeeding.

## 2. Methodology

**Figure 1:**
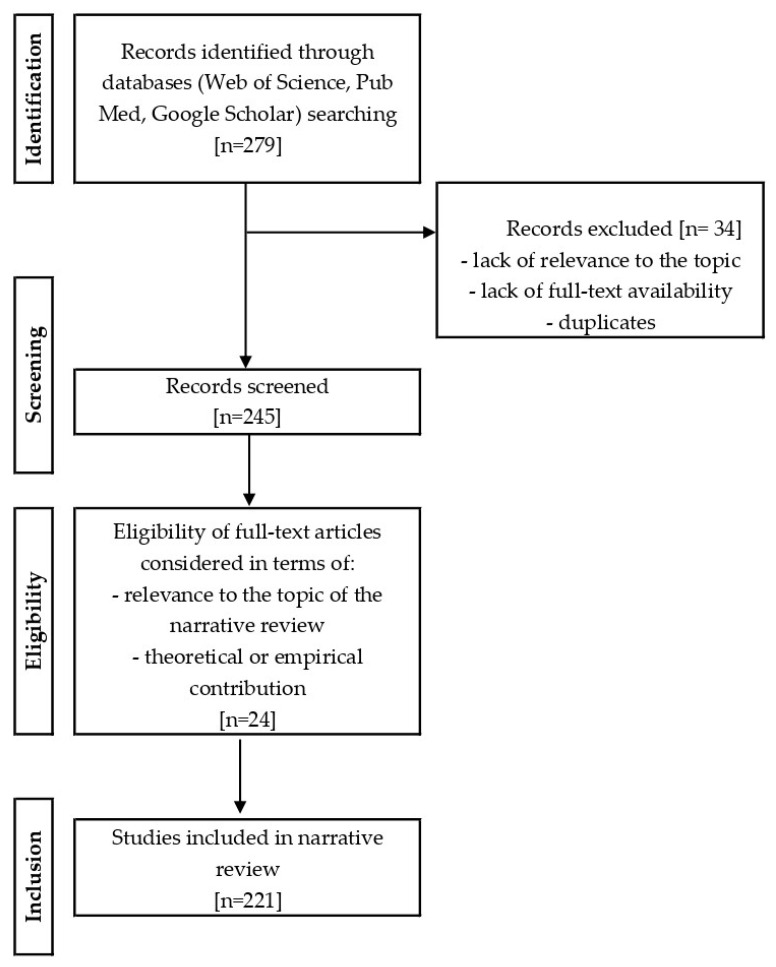
The process of selecting literature for a narrative review.

The narrative review consisted of three stages: literature search, assessment of abstracts, and analysis of full-text publications. A literature review was conducted using the Web of Science, PubMed, and Google Scholar databases. Articles published between 2000 and 2025 were analyzed. The inclusion criteria were articles published in English or Polish addressing the topic of trauma and early experiences affecting breastfeeding. Articles were searched using keywords and their variants, such as “breastfeeding” OR “lactation” AND “trauma” OR “adverse childhood experiences” OR “maternal mental health” OR “postpartum depression” OR “post-traumatic stress disorder” OR “PTSD” OR “maternal trauma” OR “birth trauma” OR “maternal psychological distress” OR “early life stress” OR “childhood abuse” OR “maternal well-being” OR “traumatic experiences in adulthood” OR “perinatal mental health” AND “mother-infant bonding” AND “psychosocial support for breastfeeding” OR “partner support for breastfeeding” OR “workplace support for breastfeeding”. Publications that did not meet the inclusion criteria were excluded from the review. The literature review included English-language articles (96.94%, *n* = 220) and Polish-language article (0.44%, *n* = 1). In addition to scientific articles, the review also included selected documents and materials from reliable institutional sources, such as the WHO and UNICEF (2.62%, *n* = 7). These materials included current guidelines, reports, and expert opinions on breastfeeding and maternal mental health. After a thorough search of the literature, the abstracts of all articles were analyzed to confirm their relevance to the topic of the article, i.e., the broadly understood impact of trauma on breastfeeding. Following analysis of the identified publications, 221 articles were selected for review. About 90% of the studies published within the previous ten years (2015–2025) were considered in review. The literature selection process is presented graphically in Figure 1. Materials from international organizations (WHO and UNICEF websites) were searched for specifically and were not included in the research selection block diagram, which refers only to scientific publications.

## 3. Childhood Trauma and Breastfeeding

### 3.1. Types of Childhood Trauma and Their Impact on Emotional Development

**Figure 2:**
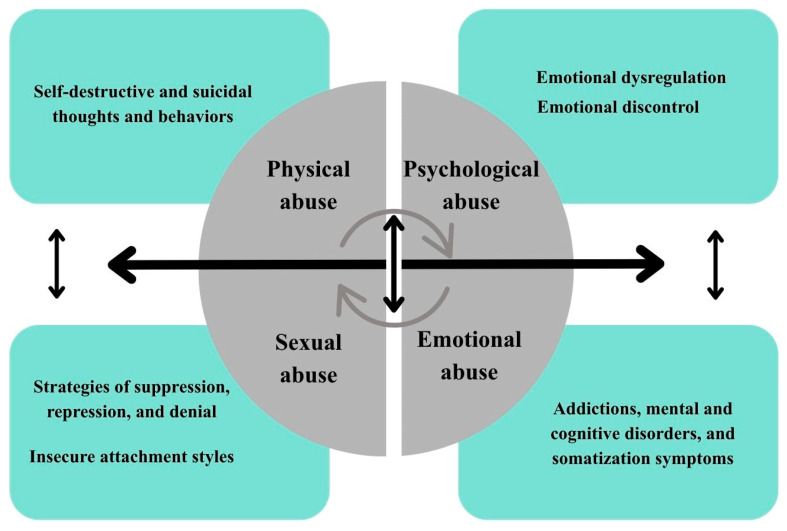
The impact of trauma on strategies and mechanisms involving the circular cycle. Explanations: Bidirectional arrows indicate reciprocal relationships between types of abuse and psychological outcomes. Circular arrows represent reinforcing cycles among different forms of abuse and their consequences.

Trauma in the current classification of the International Classification of Diseases (ICD-11) and the Diagnostic and Statistical Manual of Mental Disorders-5 (DSM-5) is the experience of an event or situation that threatens the psychophysical integrity of an individual and is highly threatening or catastrophic in nature, any situation that triggers a real threat of death or danger related to the loss of life and health, the experience of sexual abuse, the experience of serious injuries experiences, experiencing a disaster or witnessing such situations, constitutes a dimension of trauma/traumatic stress [29] (Figure 2). The literature distinguishes between different types of traumas: single trauma (e.g., after an accident), classified as PTSD (Post-Traumatic Stress Disorder), developmental trauma resulting from early childhood trauma, and relational trauma associated with repeated negative experiences in relationships. Although these situations may seem less harmful, their chronic nature can lead to serious psychophysical disorders. Childhood trauma refers to a child’s exposure to physical or emotional abuse, sexual abuse or exploitation, neglect, or other harmful experiences during development, leading to potential or actual harm to their physical and mental health, dignity, and development [30,31].

There is a strong correlation between adverse childhood experiences (ACEs) and emotional dysregulation due to the disruption of the normal bond that should be formed in parent-child interaction [32]. ACEs are reflected in emotional dysregulation due to the disruption of the normal bond that should be formed in parent-child interaction [32]. The type of attachment and relationship builds a permanent pattern in subsequent stages of development and is a lasting foundation in adult life. Early interactions with caregivers affect emotional, cognitive, spiritual, and behavioral resources, and any irregularities, neglect, or abuse lead to abnormal attachment patterns, which in turn affect emotion regulation [33]. People who have experienced abuse and neglect as children experience difficulties in regulating their emotions, resulting, for example, in abnormal emotional responses [34]. Cheng and Langevin [35] pointed out that emotional abuse increased sensitivity and alertness to detect and recognize emotions such as sadness, fear, and anger. Another important finding is that physical neglect experienced in childhood was associated in adulthood with impulsive, uncontrolled behavior and difficulties in focusing on tasks, while physical abuse experienced during developmental periods was associated with a significantly reduced perception of fear [35]. Current data indicate the global nature of the problem. According to WHO estimates, up to one billion children aged 2 to 17 have experienced physical, sexual, emotional, or neglectful abuse [36]. More than half of the world’s children have been victims of at least one form of violence in the past year, with significant differences observed between regions [37]. The consequences of mental and physical pain permeate an individual’s entire life, revealing themselves at various stages in the form of the effects of violence and abuse experienced. Research emphasizes the impact of trauma experienced during developmental periods on later difficulties in expressing emotions appropriately and an inability to manage emotional arousal. Emotional dysregulation leads to the suppression of feelings and an inappropriate response to stress, which can result in the development of anxiety disorders and depression. People struggling with such difficulties often internalize their emotions and tension, which contributes to the occurrence of psychosomatic disorders [38,39]. Downey and Crummy [40] studied coping strategies for trauma, such as denial, alcohol abuse, and isolation understood as a form of self-isolation. Authors also analyzed symptoms of anxiety, depression, sleep disorders, and self-esteem in adults to assess the impact of trauma on individual functioning. The results indicate the presence of compensatory mechanisms for traumatic experiences, such as drug and alcohol addiction, a distorted self-image, and strategies of repression and denial of the trauma experienced [40]. Dagnino et al. [41] conducted a study analyzing the relationships between different types of ACEs—physical, emotional, sexual, and physical and emotional neglect—and specific measures of emotional dysregulation, such as emotional dyscontrol, inattention, disorientation, and feelings of rejection. The study was conducted in a clinical group of adult patients with severe depressive disorder. The results confirmed the relationship between traumatic experiences and the formation of patterns and strategies of functioning in adult life [41]. The researchers also analyzed variables affecting emotional dysregulation, such as emotional dyscontrol, inattention, disorientation, feelings of rejection, and daily functional impairments. These factors are related to experiences that cause suffering and pain, such as emotional, physical, and sexual abuse or neglect. The results of the study showed that emotional abuse correlated with three dimensions of emotional dysregulation: (1) emotional distraction—difficulties in controlling impulses in stressful situations, (2) daily involvement (interference)—problems with taking goal-oriented actions in the face of negative emotions, (3) emotional inattention—lack of awareness of one’s own emotional reactions [41]. Abuse in childhood is a significant risk factor for self-aggressive behaviors and thoughts, as well as attempted and complete suicides because of traumatic experiences. The data obtained indicate that the prevalence of self-destructive thoughts and behaviors throughout life ranges from 16% to 22% and that they are more common among women than among men [42,43]. Chia et al. [44] proved in their meta-analysis that physical, emotional, and sexual abuse in childhood has a comparable impact on suicidal behavior and self-aggressive, self-destructive thoughts. The authors emphasized that each subtype of childhood abuse results in self-destructive and suicidal behaviors and thoughts. The researchers also proved that emotional neglect has a similar impact to physical neglect on predicting self-destructive thoughts and behaviors [44]. Table 1 summarizes the types of childhood trauma discussed and their impact on psychological mechanisms and behaviors in adulthood. The mechanisms described, such as emotional dysregulation, attachment insecurity, and increased reactivity to stress, are common pathways of trauma that can also be observed in traumatic experiences occurring in later stages of life.

### 3.2. The Impact of Childhood Trauma on Self-Efficacy and Self-Confidence in the Role of Mother

A woman’s understanding of herself as a mother and her attitude towards breastfeeding is one of the areas of adaptation to parenthood and is reflected in the development of the newborn, as well as in the later stages of the child’s and mother’s life. The perception of oneself as a mother and the perception of the “me-mother” and “me-mother-my child” in the context of breastfeeding influence the formation of the image of the mother and child. This process promotes the development of an emotional bond between them, which in turn supports the development of the ability to regulate emotions [45]. The approach to the role of a parent and the ability to adapt to it are an important element of adaptation to the multidimensional area of parenthood. This process includes the ability to cope with stressors in the cycle of shaping individual parental identity and building a unique emotional bond with the child [46]. Awareness of one’s own effectiveness affects the mother’s well-being and is linked to the quality of caregiving interactions with the child [47]. Researchers indicate that a high level of maternal effectiveness acts as a preventive factor minimizing the effects of pre- and postpartum depression [48,49]. Self-efficacy is the main variable in the initiation and continuation of breastfeeding, as well as in the experience and process of breastfeeding [49,50]. Parental self-efficacy is a mother’s belief in her ability to effectively cope with the demands of parenthood [51]. Confidence in the role of mother is a broader construct, encompassing both self-esteem and an emotional sense of competence in relation to the child. The mechanisms by which trauma affects functioning in the role of mother include variables through which childhood trauma can reduce feelings of efficacy and confidence [52].

Motherhood without a solid developmental foundation is associated with difficulties in fulfilling the role of a mother and a low sense of efficacy. According to the theory of parental self-efficacy, this suggests that traumatic childhood experiences can make it difficult to find one’s place as a parent [53]. The transition to motherhood is a process that involves many challenges, including adapting to new social and emotional roles. Women who become mothers often must face new duties, responsibilities, and social expectations that may be significantly different from those they have known before. In this context, self-efficacy theory is crucial to understanding how mothers’ beliefs about their abilities can shape their experiences of motherhood, referring to an individual’s belief in their ability to achieve specific tasks or goals. This theory illustrates the challenge of becoming a mother as a transition into motherhood with acquired patterns, beliefs, and experiences [54,55]. A study by Bentlay and Zamir [56] examined whether parental self-efficacy accounts for the relationship between childhood maltreatment and parental stress in mothers transitioning to motherhood. The study involved women giving birth for the first time who had children up to two years old. The results showed that experiencing abuse in childhood was associated with higher parental stress, and lower self-efficacy mediated this relationship. Mothers with childhood trauma were more likely to feel less competent as parents, which increased their stress. The study highlights the role of negative beliefs about parenting in the functioning of mothers after experiencing violence [56]. In turn, a Turkish study of mothers with children aged 0 to 6 months showed that cognitive flexibility and various contextual and personal factors can mitigate the negative impact of childhood trauma and insecure attachments [57]. The mother’s childhood trauma is a positive predictor of problematic behavior in children and confirms the theory of intergenerational transmission of trauma. The concept of the theory assumes that the trauma experienced by individuals has a negative impact on them and can also be transferred to subsequent generations [58].

It has been suggested that experiences of physical, psychological, and sexual abuse in childhood may affect the quality of the bond between mother and child. Studies show that in the case of psychological abuse, this relationship is fully mediated by symptoms of postpartum depression and low parental efficacy. Postpartum depression has also been found to be an independent factor contributing to the weakening of the bond. However, no correlation was found between sexual abuse and the variables studied, which may be due to the difficulty in disclosing such experiences. The results highlight the negative impact of psychological abuse in childhood on the mother-child relationship [59].

However, it should be noted that significant studies on childhood trauma are based on retrospective data, which may involve the risk of memory distortion and subjective interpretation of experiences [60]. This limitation should be considered when interpreting the results obtained.

### 3.3. Breastfeeding Difficulties Resulting from Previous Trauma

Emotional dysregulation and stress in women who have experienced childhood trauma often result in increased stress reactivity and difficulties in regulating emotions [61]. This results in an exaggerated sense of overwhelm in parenting situations and less confidence in one’s own parenting abilities. Negative attachment patterns as a response to early childhood trauma most often contribute to the development of avoidant or disorganized attachment patterns [62]. Mothers with this attachment style may have difficulty interpreting their child’s signals, which undermines their confidence and increases their fear of failure as a parent [63].

Low self-esteem extends into the parental sphere—mothers perceive themselves as “not good enough,” which directly reduces their sense of parental efficacy [64]. Childhood trauma increases the risk of anxiety disorders, depression, and post-traumatic symptoms in the perinatal period [65]. These symptoms negatively affect the perception of one’s own parenting skills and the ability to build a secure bond with the child. Women with a history of childhood trauma reported lower parental efficacy and higher levels of maternal stress. Mothers who experienced abuse in childhood were more likely to report uncertainty in responding to their child’s needs; they also showed higher levels of attachment based on fear [66,67]. Mothers with high levels of anxiety and anxious attachment or relational trauma may experience somatic tension during physical closeness with their child while feeding and close contact with their child [68]. Studies examining the relationship between maternal mental health and child development showed that levels of anxiety and depression in mothers are associated with problems in their relationships with their children, including difficulties in establishing closeness [69,70].

## 4. Traumatic Experiences in Adulthood

Traumatic experiences in adulthood often result from single, serious life events, such as interpersonal violence, accidents, or disasters, and can lead to symptoms of anxiety, depression, and other emotional difficulties, which means that their impact on mental functioning is often situational [71].

### 4.1. The Impact of Intimate Partner Violence on Breastfeeding

Intimate Partner Violence (IPV) is a phenomenon that has a significant impact on the health of women and their children [72]. It has been shown that experiencing violence during pregnancy and the postpartum period can negatively affect the initiation and continuation of breastfeeding, which in turn has long-term consequences for the health of the mother and child [73]. The WHO defines intimate partner violence as: “any behavior by a current or former partner in the context of marriage, cohabitation, or any other formal or informal relationship that causes physical, sexual, or psychological harm.” Statistics estimate that 641–753 million women aged 15 and older have experienced IPV at least once in their lifetime [74].

Available studies indicate that women exposed to IPV are less likely to start breastfeeding or breastfeed for a significantly shorter period [75,76]. Furthermore, it has been pointed out that women who have experienced IPV are more likely to doubt their parenting skills and breastfeeding abilities [77].

Continuing to breastfeed for up to 6 weeks after giving birth may mitigate the negative effects of prenatal violence on an infant’s temperament [78]. Research has shown that children of mothers who have experienced IPV may exhibit greater emotional and behavioral problems, underscoring the importance of breastfeeding support for the mental health of both mothers and children [79]. The impact of IPV on the health of mothers and infants confirms that experiencing violence is associated with a higher risk of health problems such as depression, anxiety, and reduced ability to breastfeed [80,81].

### 4.2. The Impact of Difficult Perinatal Experiences on Emotions and Breastfeeding

Difficult perinatal experiences, such as miscarriage or traumatic childbirth, can significantly affect a woman’s emotional functioning after the birth of her child [82]. High levels of stress, anxiety, or feelings of loss often also affect the course and experience of breastfeeding. Although the mechanisms differ from childhood trauma, similar challenges in responding to stress can affect the feeding process. Understanding these relationships is crucial to providing mothers with adequate psychological and lactation support [27].

Miscarriage, defined as the loss of a pregnancy before 20 weeks, affects approximately 12–26% of recognized pregnancies worldwide [83]. The effects of miscarriage are not only physical but also psychological, affecting women’s emotional well-being and their subsequent experiences with breastfeeding. The experience of miscarriage often leads to intense emotions such as sadness, anger, guilt, and anxiety [84]. Many women after a miscarriage struggle with symptoms of depression and anxiety that can last for a long time. It is reported that about 11% of women experience moderate to severe depression after a miscarriage. The psychological trauma associated with miscarriage can make women more susceptible to anxiety and depression in subsequent pregnancies [85], which in turn can interfere with lactation.

There is a significant association between birth stress, perception of birth as traumatic, symptoms of post-traumatic stress disorder, and difficulties in establishing exclusive breastfeeding [86]. The relationship between unsuccessful or lack of breastfeeding and birth trauma and PTSD is complex and interactive [87]. Some mothers who have experienced a difficult birth may perceive breastfeeding as a psychological trauma that reminds them of the hardship and pain [87]. Research shows that traumatic childbirth, which may include intense pain, unexpected medical interventions, and lack of emotional support, is strongly correlated with the onset of PTSD in mothers [88]. Women who experience PTSD after childbirth often report difficulties in forming emotional bonds with their newborns, which negatively affects their decisions about breastfeeding [89]. Breastfeeding after a traumatic perinatal experience can trigger memories of the traumatic birth and, as a result, become discouraging for a mother trying to avoid psychological distress [87]. On the other hand, some mothers see breastfeeding as another stage of childbirth. Therefore, the lack of breastfeeding can intensify feelings of failure after a difficult birth, leading to sadness and frustration [87]. In this case, breastfeeding can be a healing process after the trauma of childbirth and strengthen their role as a mother [90].

Hormones play an important role in the development of depressive symptoms while also affecting lactation, bonding with the baby, and stress responses [91]. Oxytocin helps regulate cortisol levels, supporting the body’s rapid return to balance after stress, and breastfeeding women show reduced adrenocorticotropic hormone (ACTH) and glucose responses [92]. Mothers with PTSD have higher cortisol levels in the postpartum period, which may be associated with hormonal dysregulation [93]. Higher oxytocin levels typically support the mother-infant bond, but this effect may be attenuated in women with prior trauma; after a traumatic birth, oxytocin may act adaptively, reducing stress and facilitating feeding [91,94].

Traumatic birth experiences increase the incidence of PTSD. They may be associated with changes to the birth plan, complications during labor, emergency cesarean sections, or verbal or psychoemotional abuse by healthcare professionals [95]. Supporting women during childbirth and breastfeeding can alleviate or reduce the severity of mental disorders in the postpartum period [87].

## 5. Mental Disorders and Breastfeeding

### 5.1. Postpartum Depression

Postpartum depression is a common mental health condition that affects many mothers around the world. It is associated with persistent sadness and reduced sense of well-being and significantly affects the mother’s daily functioning and mood. It is estimated that postpartum depression affects approximately 10–20% of women and therefore constitutes a serious public health problem [96]. The postpartum period is a special time for mothers, during which proper care is essential to support their physical, emotional, and mental well-being [97]. The high incidence of postpartum depression indicates the need to raise awareness in this area and highlights the need to support women in the postpartum period. It is essential that medical staff, policymakers, and the public fully recognize the prevalence of this problem and take action to ensure adequate support and accessible forms of assistance for mothers who experience it [98,99].

Risk factors for postpartum depression primarily include genetic predisposition and a history of mental disorders. Women who have experienced mental health problems in the past are more likely to develop depressive symptoms after giving birth. Mental disorders in the family may suggest a genetic predisposition and a lasting influence of the biological mechanisms underlying mood disorders [100,101]. Rapid hormonal changes occurring in the postpartum period, primarily involving a decrease in estrogen and progesterone levels, are significant in the development of postpartum depression. This sudden change can disrupt the balance of neurotransmitters responsible for regulating emotions and mood. Combined with sleep deprivation, stress, and physical exhaustion, hormonal fluctuations can be a significant mechanism contributing to the onset of postpartum depression [102]. Insufficient emotional and practical support from a partner, family, or friends is one of the key factors contributing to the development of postpartum depression. The lack of a support network can exacerbate feelings of loneliness, overload, and lack of understanding, which increases the emotional vulnerability of young mothers [11]. In addition, high levels of stress or sudden life changes can be a significant trigger for depressive symptoms [103].

Postpartum depression significantly interferes with the development of the bond between mother and child. Women experiencing symptoms of depression often have difficulty establishing a strong and secure emotional bond with their child [104]. These mechanisms may include impaired emotion regulation, reduced oxytocin secretion, increased cortisol levels, and difficulty in reading the baby’s signals, which together may limit the effectiveness of breastfeeding [27]. This can manifest itself in a lack of touch, infrequent eye contact, and reduced expressions of joy and delight towards their child. These behaviors are most often the result of the mothers’ emotional problems, including chronic sadness, fatigue, and anxiety caused by postpartum depression [104]. Another characteristic symptom of postpartum depression is inconsistent reactions, manifested by difficulties in consistently paying attention to the child’s needs and responding quickly enough to their signals. This can lead to feelings of anxiety and confusion in the infant [105]. Disturbances in the mother-child attachment relationship can lead to long-term consequences in the socio-emotional development of the child [106]. As a result of these difficulties, insecure attachment patterns may develop, manifesting as ambivalence, avoidance, or resistance toward the mother, which may increase the risk of emotional and behavioral problems later in the child’s life [107,108]. That is why it is so important to recognize the impact of postpartum depression on the mother-child relationship and to implement appropriate forms of support and therapy. Early diagnosis, intervention, and targeted therapeutic measures can help mothers build a safer, more supportive bond with their child, which promotes harmonious emotional development and well-being for both [109].

The bi-directional relationship between maternal mental health and poor breastfeeding outcomes has been well documented. Mood disorders can affect the psychophysiological mechanisms of lactation, such as decreased oxytocin secretion, increased stress response, and difficulties in regulating emotions during feeding, which may limit the duration and quality of breastfeeding [6,27]. Women with symptoms of depression or anxiety are less likely to initiate breastfeeding, more likely to discontinue it early, and encounter more difficulties in breastfeeding compared to mothers without mood disorders [26,110]. At the same time, breastfeeding problems, including mastitis, difficulties with proper latching, or severe pain during feeding, can contribute to the development of postpartum depression [26,111]. Postpartum depression contributes to lower motivation, low energy levels, and a lack of desire to engage in breastfeeding [13]. These symptoms may interact with cognitive-emotional mechanisms, such as reduced confidence in one’s role as a mother, feelings of guilt, and stress, which further hinder the continuation of breastfeeding [112]. In addition, it should be noted that some medications used to treat depression pass into breast milk and may affect the infant. As a result, many women face a difficult dilemma regarding whether to continue pharmacological treatment while breastfeeding [113]. Many available studies indicate the impact of depressive symptoms in mothers on breastfeeding, manifested, among other things, by problems with breastfeeding or discontinuation of breastfeeding [114,115,116]. A systematic review by Slomian et al. [117] showed that in almost all the studies presented, postpartum depression was associated with a shorter period of breastfeeding. Although postpartum depression is recognized as a risk factor for premature cessation of breastfeeding, negative experiences related to breastfeeding may increase the risk of its occurrence [118]. This disorder can negatively affect the mother’s self-esteem and cognitive functions, making it difficult to establish adequate interactions with the newborn, which contributes to difficulties in breastfeeding.

### 5.2. Anxiety Disorders

According to available statistical data, women are approximately 60% more likely to develop anxiety disorders than men. During pregnancy, women very often experience anxiety, which, if left untreated, can persist for a long time. The impact of postpartum anxiety is multifaceted and can be manifested, including impaired maternal functioning, causing anxiety, and disrupting the mother-child bond [119]. These mechanisms include impaired emotion regulation, increased cortisol levels, and the resulting difficulties in interpreting the infant’s cues, which may contribute to reduced breastfeeding effectiveness [120].

**Figure 3:**
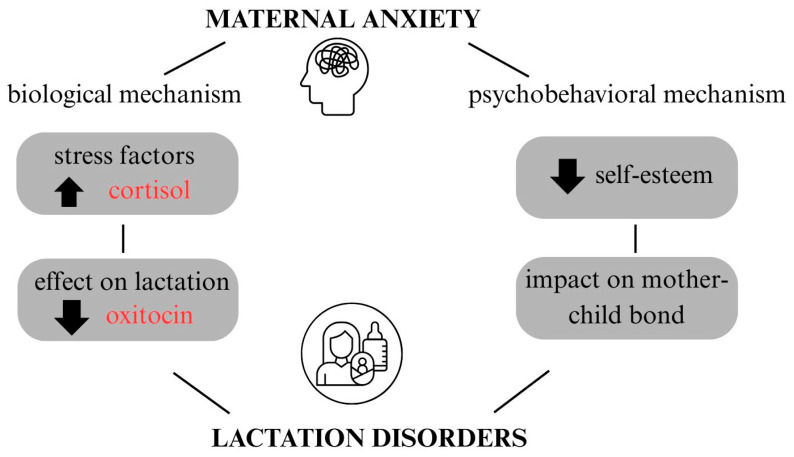
The impact of anxiety on breastfeeding. Explanations: Upward and downward arrows represent relative increases and decreases in the levels of the illustrated variables.

Anxiety initiated during pregnancy is associated with subsequent difficulties in breastfeeding [27]. Anxiety can be a barrier to optimal breastfeeding in women who experience it. Two mechanisms through which anxiety affects breastfeeding have been identified: biological and psychobehavioral [121] (Figure 3). Biologically, anxiety is associated with disturbances in oxytocin secretion and dysregulation of the hypothalamic–pituitary–adrenal axis, which can interfere with the milk ejection reflex and, as a result, hinder the feeding process. On the other hand, the psychobehavioral mechanism refers to the impact of anxiety on the mother’s emotional state lowers her sense of self-efficacy and self-worth, which negatively affects mother-child interactions and the breastfeeding experience itself. As a result, although prenatal anxiety does not necessarily directly interfere with lactation itself, it can significantly affect the psychological aspect of feeding, reducing the mother’s confidence and satisfaction with breastfeeding [119,121]. Neupane et al. [122] showed that the co-occurrence of anxiety and postpartum depression affects exclusive breastfeeding and significantly reduces the chances of maintaining it for the first six months of a child’s life. Early diagnosis and psychological support can reduce the negative effects of mood disorders and anxiety by helping with emotional regulation, stabilizing stress levels, and strengthening the mother-child relationship [123,124].

### 5.3. Obsessive–Compulsive Disorder

The postpartum period is susceptible to obsessive-compulsive disorders (OCD) in women, including intrusive, persistent thoughts or repetitive behaviors that cause a high level of stress [125]. It is reported that approximately 0.7–2.7% of women after childbirth may suffer from OCD. The main reason for its occurrence is hormonal changes after childbirth, including a decrease in estrogen levels and an associated decrease in serotonin, combined with factors such as fatigue and poor sleep [126]. These neurohormonal changes can affect emotion regulation and stress levels, which indirectly influence breastfeeding practices [127].

In women with OCD in the postpartum period, intrusive thoughts often relate to fears of accidental harm or loss and stem from anxiety related to sudden infant death syndrome. As a result, mothers may modify their behavior towards their infant—avoiding certain situations or controlling them excessively. This disorder causes many women to perceive their parenthood as difficult and less joyful, especially when it comes to the freedom to express their feelings [125]. Such behaviors may result from both cognitive-emotional mechanisms (high levels of anxiety, intrusive thoughts) and psychophysiological mechanisms (excessive activation of the hypothalamic–pituitary–adrenal (HPA) axis and oxytocin disorders), which together influence the breastfeeding experience [128,129].

There are few studies available showing a relationship between the occurrence of OCD and breastfeeding. Challacombe et al. [130] indicated that women diagnosed with obsessive–compulsive disorder were less likely to breastfeed within 6 months after giving birth. The authors also hypothesized that the presence of obsessive–compulsive disorder may cause lower self-efficacy in the role of mother, resulting in feeding disorders. However, Philips et al. [125] pointed out that after considering the symptoms of depression, the impact of OCD symptoms on the Breastfeeding Self-Efficacy Scale-Short Form (BSES-SF) score was weakened and the confidence interval for this effect included zero, suggesting that it is difficult to separate the effects of obsessive–compulsive disorder from coexisting depression. Philips et al. [125] also pointed out that women with severe symptoms of OCD may express milk much more frequently, up to 12 times a day, which may interfere with their daily self-care behaviors. In addition, anxiety-related feeding rituals were observed, such as washing baby bottles and dishes or repeatedly counting and organizing stored breast milk. These findings suggest that excessive pumping and repetitive behaviors may reflect symptoms of postpartum OCD. Such behaviors may reflect both psychological symptoms of OCD (compulsive rituals, anxiety) and psychophysiological effects of stress, affecting the duration, quality, and experience of breastfeeding.

### 5.4. Eating Disorders

Pregnancy and early motherhood, and the associated changes in women’s appearance, can negatively affect their self-perception and lower their self-esteem. Many women report dissatisfaction with their appearance after giving birth, regardless of whether they have an eating disorder [131]. It is assumed that these changes are more difficult to cope with and accept for women who have experienced eating disorders or have concerns about their appearance. These women are more likely to experience body dissatisfaction, low self-esteem, perfectionist tendencies, and feelings of shame, which can make it difficult to adapt to motherhood and increase the risk of postpartum anxiety and depression [132].

Research indicates that women with current or previous eating disorders may encounter difficulties in breastfeeding, and earlier cessation of breastfeeding and shorter breastfeeding duration are also observed [133]. In addition, these women experience more emotional problems related to breastfeeding and conflict between priorities regarding their own health and the well-being of their child [132,134]. Available studies indicate that mothers with eating disorders were more likely to stop breastfeeding within the first six months after giving birth, unlike women without eating disorders [135].

Mothers with eating disorders may have different attitudes towards their children. Women with bulimia may fear that their children will overeat, which may lead to prolonged breastfeeding to avoid contact with solid foods. In the case of women with anorexia, there is a risk of extreme malnutrition and insufficient milk production and thus premature cessation of breastfeeding [133]. On the other hand, it has been pointed out that obese women are less likely to start breastfeeding and breastfeed for shorter periods of time due to mechanical difficulties in latching the baby onto the breast and discomfort related to their body shape and the resulting lack of privacy [136].

At the neurohormonal level, women with eating disorders have impaired secretion of oxytocin, which is released during breastfeeding and promotes the mother-child bond [137]. It is reported that oxytocin is responsible for regulating eating behavior and may play a role in the disease process of eating disorders. Although this mechanism is not yet fully understood, it has been suggested that the action of oxytocin may be the cause of breastfeeding disorders in women diagnosed with eating disorders [138].

In summary, depressive, anxiety, obsessive-compulsive, and eating disorders in the perinatal period have a significant impact on decisions and practices related to breastfeeding. The symptoms of these disorders can interfere with the initiation and maintenance of breastfeeding, undermine the mother’s sense of self-efficacy, and disrupt attachment bonding with the infant. It is worth noting that the impact of these disorders is bidirectional: difficulties in breastfeeding can exacerbate mental health symptoms, and existing mental health disorders can hinder successful breastfeeding [27]. Table 2 summarizes the common and specific mechanisms for each of the disorders described their impact on lactation, and clinical support. The detailed psychological, neurohormonal, and behavioral mechanisms that explain how the symptoms of these disorders affect the breastfeeding process are discussed in Section 6. Referring to these mechanisms provides a better understanding of the complex interaction between maternal mental health and breastfeeding practices and provides a basis for the development of effective clinical support and interventions.

## 6. Psychological and Psychophysiological Mechanisms in Breastfeeding

**Figure 4:**
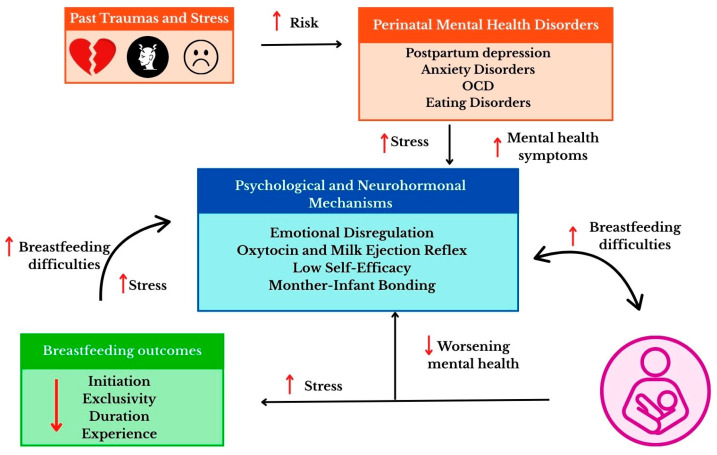
The relationship between trauma, psychological mechanisms, and breastfeeding outcomes. Explanations: Upward arrows indicate increases (e.g., levels of stress, risk, mental health symptoms, breastfeeding difficulties), whereas downward arrows indicate decreases (e.g., mental health symptoms, breastfeeding outcomes). Circular arrows represent bidirectional relationships and feedback mechanisms among the illustrated process.

Breastfeeding is a complex process involving physiological, hormonal, and psychological mechanisms. Milk production and secretion depend on a properly developed mammary gland and hormonal regulation [143]. These reactions are strongly modulated by psychological factors—stress levels, previous emotional experiences, as well as a history of trauma or anxiety and depressive disorders [6]. Figure 4 shows the interactions between trauma, mental health, and breastfeeding mechanisms.

Interference with the hypothalamic–pituitary–adrenal axis or changes in oxytocin secretion can affect lactation, hinder milk flow and weaken the feeling of closeness with the baby [144]. Understanding the interrelationships between psychological and physiological processes is therefore crucial for a trauma-informed interpretation on how previous psychological experiences—including trauma—may affect the ability to breastfeed and the mother–infant relationship.

### 6.1. Emotion Regulation

It is widely known that a mother’s emotions influence breastfeeding behavior [14]. Poor emotion regulation mechanisms among mothers can negatively affect their mental health [145]. The most studied relationships are those between the impact of postpartum depression on breastfeeding indicators [146]. Based on research by Li et al. [146], it was shown that the use of strategies involving the suppression of emotional expression was an independent risk factor hindering the initiation of breastfeeding in newborns. At the same time, the authors showed that prenatal education on lactation, provided by healthcare professionals, primarily doctors and nurses, promoted the initiation and continuation of breastfeeding. These results underscore the importance of analyzing ways of regulating emotions in women during the perinatal period and the need to tailor educational programs and lactation counseling to the individual needs of mothers. It is also pointed out that suppressing emotional expression can disrupt the natural process of emotional regulation in the postpartum period, which in turn affects the neurobiological mechanisms responsible for lactation [146]. Suppressing emotions are often associated with depressive behaviors and lower levels of life satisfaction [147]. Furthermore, these behaviors are associated with heightened symptoms of post-traumatic stress disorder, anxiety, and depression among individuals who have experienced traumatic events in the past [148]. In the case of breastfeeding women, traumatic experiences related to fear of pregnancy may influence mothers’ aspirations to breastfeed [144].

### 6.2. Neurohormonal Basis of Lactation

Lactation is a process in which physiological and emotional factors are closely intertwined [143]. The most important hormones involved in this process are prolactin and oxytocin, secreted by the pituitary gland under the control of the hypothalamus [149]. Prolactin is responsible for milk production in the mammary glands and stimulates milk production during pregnancy and after childbirth [150]. Oxytocin, on the other hand, promotes milk secretion by contracting the myoepithelial cells surrounding the alveoli of the mammary gland. Oxytocin is released when a baby suckles at the breast, but its release is also triggered in response to emotional stimuli, such as the sound of a baby crying or eye contact with the baby [151]. In addition to its physiological function, oxytocin also acts as a neuromodulator of emotions and is associated with promoting human social interactions, reducing fear and pain, and reducing physiological and psychological stress [152]. Oxytocin is responsible for the development and maintenance of the unique mother-child bond [153].

### 6.3. The Importance of the Mother-Child Bond

The emotional bond between mother and child, which develops during pregnancy and strengthens after birth, forms the basis of secure attachment. This relationship gives the child a sense of security, encourages exploration of their surroundings, and promotes the development of healthy relationships in the future [154]. Secure attachment in the postnatal period is crucial for the child’s mental, emotional, and social development. It supports emotion regulation, stress resilience, and the development of social and cognitive skills and protects against anxiety and depression [154,155]. There are factors that can hinder the development of a strong mother-child bond. These include, first and foremost, mental health problems in mothers [156]. In this state, a mother may have an impaired ability to engage in bonding with her child [157]. A life-course perspective highlights how traumatic experiences earlier in life and the resulting post-traumatic stress disorder can impair mothers’ mental health, hindering their ability to build a sense of safety and trust in their relationship with their child [158]. Difficulties in forming an emotional bond with an infant can affect how a woman perceives herself as a mother. A lack of closeness, difficulty in reading the child’s signals, or reduced confidence in one’s own parenting skills can lead to avoidance of physical contact, including a lack of initiation of breastfeeding. On the other hand, the experience of trauma related to breastfeeding can in turn weaken the emotional bond between mother and child [159,160], contributing to premature cessation of lactation or giving up breastfeeding in the case of subsequent children [2].

### 6.4. Stress and Its Impact on Milk Production

There is a widespread belief that stress is a factor affecting breastfeeding performance [161]. The presence of stressors, mainly related to financial problems and emotional problems in the relationship with the partner, is associated with a lower likelihood of initiating breastfeeding and a higher risk of early cessation of lactation [162]. Stress can directly affect the composition of milk. In response to short-term stressors in women, cortisol levels rise and may increase the level of a substrate necessary for the synthesis of carbohydrates and lipids, which may promote higher levels of lactose and fat in milk. Ziomkiewicz et al. [161] explain that elevated cortisol levels in response to short-term stress can temporarily mobilize the mother’s fat reserves, increasing the availability of substrates for fat synthesis in milk. However, in the case of prolonged stress, this mobilization leads to a gradual depletion of energy resources, which in turn limits the number of substrates needed for milk fat production. On the other hand, chronic stress can have more serious consequences and have a greater impact on the composition of breast milk. Prolonged tension can change eating habits—both increasing and decreasing the consumption of high-calorie foods. Since the fat content of breast milk depends on diet, such changes in energy and fat intake may affect the amount and composition of fatty acids in milk [161].

Stress affects lactation not only through metabolic changes but also at the hormonal level. Activation of the HPA axis leads to increased cortisol secretion, which can disrupt the hormonal balance necessary for normal milk production. At the same time, chronic stress lowers prolactin levels, a hormone that is key to initiating and maintaining lactation [6]. Stress factors impair the release of oxytocin, which is responsible for the milk ejection reflex and facilitates breast emptying during feeding [162]. It has been suggested that the occurrence of anxiety and depression is associated with lower levels of oxytocin in mothers during feeding [163]. In addition, chronic stress has a negative impact on the mental state of mothers, and a reduction in women’s comfort can disrupt the process of effective breastfeeding [161,164].

### 6.5. Body Image

The postpartum period often confronts women with the discrepancy between the idealized image of motherhood and the reality of everyday challenges [165]. Many women feel anxious about their bodies and appearance. The appearance of pregnant and postpartum women is often idealized by the media, which puts women under a lot of pressure and makes them particularly concerned about their weight. For many women, concerns about their body image during pregnancy are a continuation of past experiences of negative perceptions of their appearance [165]. Creating perfect appearance and behavior during pregnancy often goes hand in hand with difficulties in experiencing positive feelings during pregnancy and motherhood and pressure to rapidly regain pre-pregnancy body shape [166,167]. In addition, the breastfeeding period itself is highly idealized, which also contributes to feelings of failure among women who have been unable to breastfeed. Breastfeeding is promoted as easy and unproblematic, but it is very often associated with pain and discomfort for mothers [165]. It is pointed out that breastfeeding is subject to strong socio-cultural pressure and is closely related to the perception of a woman’s body and identity [168]. As a result, the way women interpret their bodily experiences can affect not only their mental well-being but also physiological processes such as lactation.

## 7. Social, Cultural, and Structural Contexts of Breastfeeding

**Figure 5:**
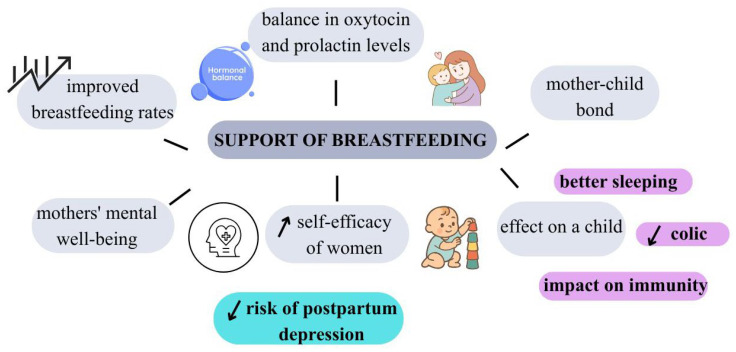
The impact of social and environmental support on breastfeeding. Explanations: Upward and downward arrows represent relative increases and decreases in the levels of the illustrated variables.

Women who have just given birth expect the most support from those closest to them—their partner, children, parents, or friends. It is widely known that when a child is born, many dynamic changes take place in the family, and all attention is focused on the newborn, while the well-being of mothers takes a back seat [169,170]. According to available research, social support plays a leading role in difficult moments for mothers, such as childbirth and the postpartum period, and supports mental health by acting as a buffer against mental health problems [171]. Conversely, a lack of social support significantly contributes to a deterioration in mothers’ mood [172] (Figure 5). The observed relationships are consistent with the socio-ecological model, which assumes that an individual’s mental health is shaped by interactions between the individual, relational, and social levels [173].

### 7.1. Support and Relationship

#### 7.1.1. Support from Close Social Network

Family members are an easily accessible and irreplaceable source of support for women. According to the socio-ecological model, support from a close social network influences breastfeeding decisions by acting at the individual, social, and structural levels [174]. The participants in the study presented by James et al. [175] received emotional, physical, and verbal support from family and friends, which can be a reliable source of support in overcoming breastfeeding difficulties. The effectiveness of support from a close social network has been confirmed in educational programs for families and partners implemented during the prenatal and postnatal periods [176]. These programs, which include practical information on lactation and strategies for supporting the mother’s mental well-being, are associated with higher rates of exclusive breastfeeding and longer duration of breastfeeding [177]. The support of loved ones during this difficult period for women is extremely important, but the values and beliefs of those providing support should be consistent with the values and needs of the mother herself [178]. It is important that support from family members does not take the form of pressure [28]. Experiences of trauma or difficult family relationships can influence how women perceive support from those around them, so it is important to adjust support to the individual needs of the mother [179]. It has been shown that the presence of a stable partner who is supportive and helps women find their way in their new role has a direct impact on the duration of breastfeeding [178]. It has been shown that actively involving partners in lactation education—through prenatal consultations, partner workshops, or educational materials aimed directly at fathers—increases their sense of competence and readiness to provide support, which translates into better lactation outcomes [180,181]. Actions such as verbal support, help with household chores, or caring for older children have a significant impact on maintaining exclusive breastfeeding [178]. Therefore, it is indicated that married women breastfeed longer than single or divorced women [182]. Research shows that married women are more likely to maintain exclusive breastfeeding after three and six months compared to unmarried women, which may be associated with greater emotional and material support [183,184]. At the same time, it should be noted that marital status often correlates with other structural factors, such as economic stability, access to healthcare, and parental leave, which also influence the ability to continue breastfeeding [185]. Furthermore, in a study by Murillo-Llorente et al. [182] showed that women who are more satisfied with breastfeeding are married. It should also be emphasized that partners, when unable to help women with lactation problems, often experience helplessness and frustration, feeling excluded from the process [186]. It is important to involve partners in breastfeeding interventions, as this can motivate them to show greater support and understanding for breastfeeding mothers [187]. Healthcare personnel need additional training in providing breastfeeding education tailored to the needs of family members so that they can support women more effectively and, if necessary, flexibly modify the way they communicate [188]. This approach not only enables more effective support for breastfeeding women but also integrates their close social network into lactation care in a way that respects cultural diversity, life experiences, and the individual needs of mothers.

#### 7.1.2. Workplace Support

The work environment, including organizational conditions and employer policies, plays an important role in continuing breastfeeding after women return to work [189]. Employers also have an indirect influence on the length of time women breastfeed. A 2020 study shows that among female doctors, only 13% of the 87% of residents who expressed a desire to breastfeed their child for 12 months achieved their goals [190]. The main reasons women give for stopping breastfeeding are insufficient access to rooms for expressing milk, lack of space to store expressed milk, and lack of support from employers and colleagues [191,192,193]. These factors show that appropriate workplace organization and support from employers and coworkers can make it easier for women to continue breastfeeding [194].

Mothers’ concerns about continuing breastfeeding also result from returning to work after maternity leave. A positive correlation has been found between the length of maternity leave and the duration of breastfeeding [195]. The differences in access to maternity leave and flexible forms of employment may thus contribute to inequalities in the ability to continue breastfeeding among women [196]. Support from management and a positive attitude from colleagues can determine decisions to continue breastfeeding and even double its duration [197,198]. It is emphasized that both employers and colleagues who have children and have more experience with breastfeeding show stronger support for breastfeeding mothers at work [199]. Tsai et al. [189] analyzed the impact of designated breastfeeding or pumping spaces, flexible pumping time, and support from coworkers on the continuation of breastfeeding after returning to work. The results indicated that encouragement from colleagues to take breaks for pumping significantly increased the likelihood of continuing to breastfeed for the first six months. It should be emphasized that pumping at work is also related to the individual characteristics of mothers. Women who have previously felt ashamed or struggled with difficult experiences related to breastfeeding may find it more difficult to adapt to expressing milk at work [200]. In such cases, a supportive and pressure-free work environment is particularly important, as it can help women adapt to expressing milk and reduce their psychological burden [201]. Some countries, including Australia, New Zealand, and Austria, have strong breastfeeding policies and offer extensive support to women in this area [200]. In turn, developing countries (Thailand, Myanmar, Laos, Indonesia) that attach great importance to promoting breastfeeding have high breastfeeding maintenance rates [200]. Similarly, in China, it has been shown that women who have access to a separate breastfeeding room at work are less likely to stop breastfeeding [202]. In Malaysia, the lack of adequate breastfeeding facilities in the workplace was associated with a higher likelihood of stopping breastfeeding [203]. These differences indicate that the effectiveness of breastfeeding support depends on the social, political, and organizational context in which working mothers operate. Women who have previous experience with expressing milk and who are more confident in using lactation spaces and breaks tend to find it easier to organize this process at work, adapt more quickly to new conditions, and maintain lactation more effectively [204]. A study conducted by Zhuang et al. [205] analyzed factors influencing attitudes toward breastfeeding women in the workplace and their self-efficacy in expressing milk. The authors noted that negative emotions towards breast milk (“ick response”) promoted stigmatization, while a sense of justice and support reduced it. In addition, it was shown that women’s self-efficacy in expressing milk at work was positively associated with a sense of fairness, which emphasizes the importance of a friendly and accepting work environment. The collected data emphasize that the work environment is an important element of the socio-ecological conditions for breastfeeding, including organizational, social, and individual factors.

#### 7.1.3. Professional Support

The support of medical staff in assisting breastfeeding mothers plays a very important role, both in initiating breastfeeding and in maintaining lactation and building positive experiences related to motherhood [186]. The functioning of healthcare institutions, including the actions of medical staff, has a significant impact on women’s experiences of breastfeeding [206]. A negative attitude on the part of hospital staff perceived by women after childbirth, including a lack of support and neutrality in decision-making, is a factor contributing to breastfeeding failure. Such experiences can be particularly difficult for women after childbirth, increasing stress and reducing their sense of self-efficacy in breastfeeding [207]. On the other hand, it has been pointed out that many healthcare professionals do not have sufficient knowledge in this area. Breastfeeding support programs include education and training for all disciplines of staff who support breastfeeding mothers. These trainings may take the form of standardized educational programs, clinical mentoring, and the implementation of uniform procedures, which promote consistency in the information provided and the quality of care [186,208]. However, it should be noted that personal experiences with breastfeeding can translate into diverse views among medical staff. In settings where breastfeeding rates have historically been low, healthcare personnel may have experienced difficulties with breastfeeding themselves, which directly influences their subsequent attitudes towards the practice. For example, in one study, only 45% of doctors and 65% of nurses believed that infants should be exclusively breastfed for the first six months of life [209]. It is important for medical staff to raise awareness among mothers and provide them with reliable information about alternatives to breastfeeding, including breast milk substitutes [210]. Education in this area should be conducted in accordance with the guidelines of the International Code of Marketing of Breast milk Substitutes to ensure that information is neutral, protect mothers from marketing pressure, and support informed decisions about feeding their babies. Exercising such caution is essential to maintaining the professionalism of medical staff and protecting the health and well-being of infants and mothers [186,210]. This indicates that support from medical staff is a central element of the socio-ecological context of breastfeeding, in which systemic, relational, and individual factors interact.

### 7.2. Cultural Norms, Stigma, and Intersectional Factors

#### 7.2.1. Social and Cultural Norms

Although breastfeeding is an individual experience, decisions about whether to start and continue breastfeeding are shaped by a woman’s own beliefs and by the explicit and implicit opinions of those around her about the benefits, difficulties, and appropriate duration of breastfeeding [24]. The strength of these norms may vary by individual. For example, a woman breastfeeding for the first time may be more sensitive to social norms than a mother who already has experience with breastfeeding or infant formula [211]. In a study by Carlin et al. [24] found that mothers were influenced by the norms operating in their social networks. Some women felt pressure to breastfeed from healthcare professionals or partners who were opposed to the use of infant formula. Other women experienced pressure from women who did not breastfeed themselves, as well as from partners who perceived the breast as a sexual symbol or feared losing their bond with the child. These opinions had a significant impact on mothers’ decisions to start and continue breastfeeding. Previous studies emphasize the important role of partners and mothers of breastfeeding women in decisions related to initiating and maintaining breastfeeding [212]. Breastfeeding women may experience shame and pressure regardless of how they feed their baby [213]. Carlin et al. [24] report that some mothers felt social pressure to breastfeed, while others emphasized acceptance from those around them when choosing infant formula instead of their own milk. However, excessive pressure can discourage breastfeeding and undermine confidence in social support. Consistent with previous studies, it was found that social norms in the workplace have a significant impact on the continuation of breastfeeding after women return to work [189,205]. Mothers employed in environments where pumping was accepted and previously practiced were less likely to perceive it as a barrier [24]. In workplaces where breastfeeding was associated with stress and difficulties for women, they were more likely to give up breastfeeding [201]. Creating supportive norms and conditions at work can therefore significantly support women in maintaining lactation after returning to work. Social norms can both support and hinder breastfeeding, depending on the degree of acceptance, pressure, and women’s previous experiences.

#### 7.2.2. Stigma, Shame, and Social Judgment

It has been pointed out that women who are unable to breastfeed for various reasons and use infant formulas are at risk of stigmatization [214]. Often, the inability to breastfeed causes women to feel guilty and self-stigmatized, which has a significant impact on their mental health [28]. A common stereotype is the stigmatization of mothers based on their age. Young mothers are perceived as inferior and judged as immature, unemployed, uneducated, lacking parenting skills, and as women with low professional potential [215]. Young mothers also have unpleasant experiences related to their treatment by healthcare professionals, including disregard, contempt, and discrimination [216,217]. Mothers also often face stigmatization related to breastfeeding practices. It is reported that, according to community opinion, “good motherhood” is strongly associated with breastfeeding. In turn, feeding infant formulas is associated with stigmatization, as it causes some mothers to feel guilt, shame, and social judgment [218]. The study by Fallon et al. [219] indicated that mothers who fed their children infant formula experienced feelings of guilt and were stigmatized for choosing a feeding method other than breastfeeding. A qualitative study conducted by Thomson et al. [213] showed that both breastfeeding and non-breastfeeding mothers experience feelings of shame related to their decisions about infant feeding. This shame is often internalized, meaning that women assimilate social norms and expectations of “good motherhood” and apply them to their own choices, even in the absence of direct criticism from those around them [213]. Similarly, a study by Russel et al. [220] showed that mothers, regardless of their feeding method, experienced comparable levels of shame, but mothers who fed their babies with infant formula or used mixed feeding reported higher levels of guilt than mothers who breastfed. Guilt was associated with shorter duration of exclusive breastfeeding and less willingness to continue breastfeeding. Stigmatization of mothers can negatively affect their mental well-being and relationships with the healthcare system, highlighting the importance of supportive communication [28,213].

#### 7.2.3. Cultural, Structural, and Intersectional Inequalities

Inequalities in the initiation and continuation of breastfeeding are strongly linked to structural and cultural factors that shape women’s everyday living conditions. These factors often overlap, creating complex barriers that go beyond the individual capabilities and motivations of mothers [221,222].

Research indicates that African American women, despite receiving information promoting breastfeeding, encounter significant obstacles in practice, resulting from, among other things, workplace restrictions, limited access to electric breast pumps, shorter maternity leave, and cultural norms and social expectations within their family and local networks [223]. In Bangladesh, several barriers at the individual, social, and systemic levels affect breastfeeding practices for infants and young children. Support for mothers may include strengthening education during interactions with medical staff and involving fathers and other loved ones in breastfeeding discussions, which helps to overcome existing constraints [224].

At the same time, community support, including peer groups and peer support, significantly increases the likelihood of breastfeeding in these populations, indicating that effective promotional strategies should combine educational activities that take into account the socio-cultural context with the creation of a supportive structural environment [225]. In the context of breastfeeding research, it is worth noting the intersectional approach, which allows us to analyze how different social factors—such as gender, race, social class, and migration status—intersect and influence mothers’ experiences. Based on existing research, it has been suggested that taking multiple social dimensions into account can reveal complex mechanisms that support or limit breastfeeding practices. Some studies on the relationship between various adverse social conditions and breastfeeding already use an intersectional approach, suggesting that analyzing intersecting social inequalities can provide a deeper understanding of the barriers and opportunities for supporting mothers [226].

From a practical perspective, these findings indicate that effective strategies to support breastfeeding should combine educational activities with the creation of supportive structural conditions and support at the local community level. Such an approach can help reduce inequalities and increase the availability of support for mothers in different cultural and social contexts [227,228].

## 8. Strengths, Limitations and Future Studies

The study has several significant strengths, primarily providing new insights into mothers’ experiences of breastfeeding through a trauma-informed lens, as the literature on this subject is limited and there has been no attempt to compile this data into a single coherent work. At the same time, study has its limitations. The review had a narrative character, which meant that there was no standardized research protocol or formal registration, which may limit the repeatability and generalizability of the results. In addition, the selection of literature was subjective, which may affect the full representativeness of the conclusions presented.

In terms of future research, it is worth considering conducting a systematic review, which would allow for a more rigorous analysis of the available data. Furthermore, the results suggest the possibility of investigating how social and structural factors, such as racism, deep-rooted poverty, or other forms of discrimination, influence decisions about initiating breastfeeding, its duration, and mothers’ experiences. Such factors may contribute to traumatic experiences; therefore, it is worth suggesting future research that combines the analysis of discrimination and trauma to better understand their interrelationships and develop more effective support strategies. In addition, future research should expand the use of an intersectional approach to breastfeeding, e.g., by analyzing how various social and economic factors interact and influence mothers’ decisions and access to support. The work of Bartkowski et al. [226] indicates that such analyses can reveal complex relationships that are difficult to capture when examining individual factors. It is possible to develop a “traumatization matrix” to examine intersecting forms of trauma in the context of mothers’ experiences and breastfeeding, which is a promising direction for future research.

## 9. Conclusions

The presented literature review indicates that mothers’ previous traumatic experiences, both in childhood and adulthood, have a significant impact on the mother-child relationship and the breastfeeding process. Traumatic childhood experiences, such as physical, emotional, or sexual abuse; neglect; or disturbed relationships with caregivers, can lead to low self-esteem, difficulties in bonding with the child, and fear of intimacy, which hinders the initiation and continuation of breastfeeding. Traumatic experiences in adulthood, including intimate partner violence or traumatic childbirth, can further increase the risk of breastfeeding difficulties by exacerbating stress, anxiety, and depressive symptoms and undermining feelings of parental competence.

The socio-cultural context plays an important role in the breastfeeding process. The presence of support from a partner, family, and the wider community; easy access to specialist help (e.g., lactation consultants, psychologists); and acceptance of breastfeeding at work and in the social environment significantly increase the likelihood of positive breastfeeding experiences, even among women with a history of trauma. Conversely, lack of support, stigmatization, or social pressure can exacerbate breastfeeding difficulties and deepen negative psychological effects.

Based on the collected research, a standardized theoretical model can be proposed in which traumatic experiences—both from childhood and adulthood—affect breastfeeding through the mother’s mental health and psychological and psychophysiological mechanisms such as emotion regulation, HPA axis functioning, oxytocin secretion, body image, and the quality of the mother-child bond. These relationships are further modified by social, cultural, and structural contexts, including social support, cultural norms, and systemic conditions.

The understanding of the impact of childhood and adult trauma on breastfeeding, combined with consideration of the socio-cultural context, is crucial for the development of targeted interventions, supportive policies, and clinical practice. In practice, a trauma-informed approach, which combines emotional support with practical assistance for mothers, is becoming increasingly important. At the same time, it is important to create breastfeeding-friendly environments, free from stigma and excessive social pressure. Future research should focus on the mechanisms linking trauma and breastfeeding difficulties, the role of social support, and the effectiveness of psychological and lactation interventions in improving maternal well-being and breastfeeding success.

## Figures and Tables

**Table 1:** Types of childhood trauma and their impact on mechanisms and behaviors in adulthood.

Type of Childhood Trauma	Impact on Psychological Mechanisms and Behavior in Adulthood	Reference
Emotional abuse	Increased sensitivity and activity in detecting and recognizing emotions (sadness, fear, anger)	[35]
Physical neglect	Difficulty coping with impulsive behaviors	[35]
Physical abuse	Reduced perception of fear	[35]
Physical and sexual abuse	Symptoms of anxiety and depression, low self-esteem, suicidal thoughts, problematic behaviors in relationships	[40]
Emotional neglect	Creating a “false self,” hiding sensitivity, need for approval, difficulty recognizing and expressing emotions	[40]
Emotional neglect and abuse	Low self-esteem, lack of self-worth, drug and alcohol abuse to cope with difficult emotions	[40]
Physical abuse	Fearful and dismissive attachment style, difficulty trusting, problems with emotional intimacy	[41]
Emotional abuse	Difficulty regulating emotions, difficulty coping with emotions in stressful situations, limited awareness of one’s own emotional reactions	[41]
Emotional neglect and abuse	Self-destructive thoughts and behaviors, suicide attempts, self-harm	[44]
Neglect and physical abuse	Suicidal thoughts and attempts, self-harm	[44]
Sexual abuse	Suicide planning, suicidal thoughts and attempts	[44]

**Table 2:** Common and specific mechanisms of mental disorders in the perinatal period, their impact on lactation, and implications for clinical support.

Mental Disorder	Common Mechanisms	Specific Mechanisms	Impact on Breastfeeding	Clinical Support	Reference
Postpartum depression	Low self-esteem, apathy, heightened stress responses, oxytocin dysregulation	Reduced motivation, difficulties in enjoying feeding, problems in establishing mother-child bonding	Reduced initiation of feeding, shortened feeding sessions, difficulties in maintaining lactation	Cognitive-behavioral therapy, interpersonal therapy, psychodynamic therapy, pharmacotherapy (selective serotonin reuptake inhibitors—SSRIs; serotonin and norepinephrine reuptake inhibitors—SNRIs; tricyclic antidepressants—TCAs)	[26,96,102,103,104,110,139]
Anxiety disorder	Low self-esteem, excessive vigilance, stress-induced hormonal imbalance	Excessive concern for the child’s safety, avoiding breastfeeding, fear of losing control	Interference with the milk ejection reflex, feeding uncertainty, stress for mother and baby	Cognitive-behavioral therapy, breastfeeding counseling, dialectical behavior therapy, effective coping training	[27,119,121,122,140]
Obsessive–compulsive disorder	Low self-esteem, intense stress reactions	Compulsive feeding rituals, obsessive checks on the child’s hygiene and health	Decreased sense of self-efficacy as a mother, maternal stress and frustration, reduced initiation of breastfeeding	Cognitive behavioral therapy, pharmacotherapy (selective serotonin reuptake inhibitors—SSRIs)	[125,126,130,141]
Eating disorder	Low self-esteem, stress, difficulties in regulating emotions	Fear of gaining weight, restricting feeding, obsession with body image	Restricted breastfeeding, tension in the mother-child relationship, risk of child malnutrition	Cognitive behavioral therapy (CBT), guided self-help approaches, focal psychodynamic therapy, pharmacological therapies	[131,132,133,134,142]

## Data Availability

No new data were created or analyzed in this study.
